# A polydiagnostic approach to cognitive deficits in schizophrenia

**DOI:** 10.1192/j.eurpsy.2021.432

**Published:** 2021-08-13

**Authors:** A. Sánchez-Torres, G. Gil-Berrozpe, R. Lorente-Omeñaca, M. Zandio, L. Moreno-Izco, E. García De Jalón, M. Ribeiro, V. Peralta, M. Cuesta

**Affiliations:** 1 Mental Health Group, Instituto de Investigación Sanitaria de Navarra (IdISNA), Pamplona, Spain; 2 Psychiatry, Complejo Hospitalario de Navarra, Pamplona, Spain; 3 Mental Health Department, Servicio Navarro de Salud-Osasunbidea, Pamplona, Spain

**Keywords:** schizophrénia, cognition, diagnostic criteria

## Abstract

**Introduction:**

Cognitive deficits are common, clinically relevant and closely linked to poor functional outcomes in everyday functioning in patients with schizophrenia and other psychoses.

**Objectives:**

To ascertain to which extent a polydiagnostic assessment of schizophrenia is associated with clinically-derived criteria of cognitive impairment and gold-standard neuropsychological assessment.

**Methods:**

We assessed 98 patients with a psychotic disorder. We tested if patients met criteria for schizophrenia according to five diagnostic classifications: Krapelin, Bleuler, Schneider, ICD-10 and DSM-IV. Also, we applied a set of clinically-derived criteria to assess cognitive impairment associated with psychosis (CIAPs). Gold-standard neuropsychological assessment was administered, covering the cognitive domains included in the MATRICS Cognitive Battery: attention, processing speed, verbal memory, visual memory, working memory, executive function and social cognition. MANOVAs were performed to test the association between polydiagnostic and clinically-derived criteria and neuropsychological assessment.

**Results:**

**Figure:**
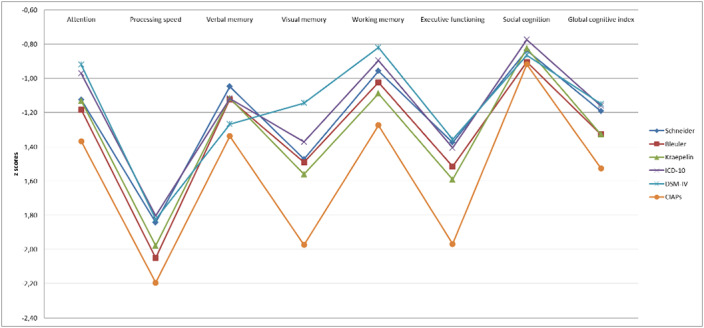

MANOVA profile analyses revealed that patients who met CIAPs criteria showed cognitive impairment in all the cognitive domains except for social cognition. Patients diagnosed with Kraepelin’s criteria showed significant differences in processing speed, visual memory, working memory and GCI. Patients fulfilling Bleuler and DSM-IV criteria showed significant deficits in processing speed and verbal memory, respectively. Schneider and ICD-10 diagnostic criteria did not reveal differences in cognition between patients who fulfilled these criteria.

**Conclusions:**

CIAPs criteria were the most accurate classifying patients with cognitive impairment, followed by Kraepelin’s criteria, which were the ones among diagnostic criteria which better differentiated patients regarding cognitive impairment. These criteria take into consideration the outcome in addition to symptoms.

**Disclosure:**

This work was supported by the Government of Navarra (grants 17/31, 18/41, 87/2014) and the Carlos III Health Institute (FEDER Funds) from the Spanish Ministry of Economy and Competitivity (14/01621 and 16/02148). Both had no further role in the study des

